# Dose-Dependent Suppression of Cytokine production from T cells by a Novel Phosphoinositide 3-Kinase Delta Inhibitor

**DOI:** 10.1038/srep30384

**Published:** 2016-07-27

**Authors:** Emily E. Way, Giraldina Trevejo-Nunez, Lawrence P. Kane, Bart H. Steiner, Kamal D. Puri, Jay K. Kolls, Kong Chen

**Affiliations:** 1 Richard King Mellon Foundation Institute for Pediatric Research, Children’s Hospital of Pittsburgh, Pittsburgh, PA, USA; 2 Department of Immunology, University of Pittsburgh, Pittsburgh, PA, USA; 3 Gilead Sciences, Inc. Seattle, WA, USA.

## Abstract

There remains a significant need for development of effective small molecules that can inhibit cytokine-mediated inflammation. Phosphoinositide 3 kinase (PI3K) is a direct upstream activator of AKT, and plays a critical role in multiple cell signaling pathways, cell cycle progression, and cell growth, and PI3K inhibitors have been approved or are in clinical development. We examined novel PI3Kdelta inhibitors, which are highly selective for the p110delta isoform of in CD3/CD28 stimulated T-cell cytokine production. *In vitro* generated CD4+ T effector cells stimulated in the presence of a PI3Kdelta inhibitor demonstrated a dose-dependent suppression of cytokines produced by Th1, Th2, and Th17 cells. This effect was T-cell intrinsic, and we observed similar effects on human PBMCs. Th17 cells expressing a constitutively activated form of AKT were resistant to PI3Kdelta inhibition, suggesting that the inhibitor is acting through AKT signaling pathways. Additionally, PI3Kdelta inhibition decreased IL-17 production *in vivo* and decreased neutrophil recruitment to the lung in a murine model of acute pulmonary inflammation. These experiments show that targeting PI3Kdelta activity can modulate T-cell cytokine production and reduce inflammation *in vivo*, suggesting that PI3Kdelta inhibition could have therapeutic potential in treating inflammatory diseases.

The immunologic pathways that lead to inflammation are extremely complex and are, in many cases, incompletely understood. Nevertheless, the components of these pathways are often therapeutic targets for drug development in the treatment of inflammatory conditions. CD4^+^ T cells play a central role in many diseases through lineage specific effector cytokine production. To date, three major effector T helper (Th) cell lineages (Th1, Th2 and Th17) have been identified according to their signature cytokines as well as lineage specific transcription factors. Inflammatory pathways involving T helper 17 (Th17) cells have become a particular therapeutic target of interest since their discovery, as these cells have been found to play a vital role in autoimmune and inflammatory conditions, as well as infection^1^,^2^. Interleukin-17 (IL-17) is a pro-inflammatory cytokine produced by several cell types, including gamma delta (γδ) T cells and the most recognized and significant of which is the CD4^+^ effector Th17 cells^3^,^4^.

Th17 cells differentiate from naïve CD4^+^ T cells upon stimulation of their T cell receptor (TCR) by antigen and exposure to TGFβ and IL-6 in mice, or to IL-1β and IL-23 in humans^5^. The TCR of Th17 cells is composed of an α and β chain dimer. During differentiation, the transcription factor Signal Transducers and Activators of Transcription (STAT)-3 stimulates activation of the critical Th17 transcription factor retinoid-related orphan receptor γt (RORγt). RORγt is known to promote expression of hallmark Th17 cytokines IL-17A, IL-17F, IL-21, and IL-22^5^. These Th17 cytokines are responsible for the recruitment of several granulopoietic factors including G-CSF, GM-CSF, as well as numerous chemokines and mucins in the process of mediating the immune response to extracellular bacteria and fungi^6^. Th17 cells play an important role in host defense against infection, but can sometimes be pathogenic when there is dysregulation in the Th17 response and its downstream effector molecules^7^,^8^,^9^. γδ T cells, by contrast, express a TCR composed of a γ and δ chain dimer and are typically thought to be involved in stimulation of the adaptive immune response by the innate immune system via driving dendritic cell maturation^10^. γδ T cells are localized to mucosal sites and their activation and production of IL-17 has been shown to be independent of IL-6, but IL-23 and IL-1β dependent^11^,^12^.

While numerous signaling pathways are involved in T cell differentiation, there has recently been focus on the role of the Phosphatidylinositol 3-Kinase (PI3K) in the differentiation of CD4^+^ T cell subsets. PI3K is an enzyme which uses cell membrane lipids to produce second messengers that are involved in numerous cellular functions including growth, survival, protein synthesis, and metabolism^13^,^14^. PI3Ks are grouped into class I, class II, and class III. Certain class IA PI3K isoforms (PI3Kα and β) are expressed ubiquitously in mammalian cells, and are composed of a 110 kDa catalytic subunit and an 85 kDa regulatory subunit^13^. The p110δ class IA PI3K is preferentially expressed in leukocytes and is crucial for CD4^+^ T cell growth and differentiation^14^,^15^,^16^,^17^,^18^,^19^.

PI3Kδ is activated by receptor tyrosine kinases including the TCR. When the TCR is stimulated by antigen presentation, PI3Kδ is activated through mechanisms which remain unclear but appear to involve TCR costimulatory and adaptor proteins^14^,^20^. Once activated, PI3Kδ generates phosphatidylinositol-3,4,5-trisphosphate (PIP3) by phosphorylating phosphatidylinositol-4,5-bisphosphate at the plasma membrane. PIP3 then recruits AKT, a serine/threonine kinase also known as Protein Kinase B, to the plasma membrane where it is phosphorylated. AKT, as a major PI3K target, plays a key role in multiple cellular processes such as glucose metabolism, apoptosis, and proliferation. PI3Kδ, in particular, plays a critical role in the differentiation process of naïve T cells into effector T helper subsets^14^,^15^,^20^, which makes it a strong candidate as a therapeutic target for drug development. There has been marked growth in the development of PI3K inhibitors, though most of these compounds have been aimed at cancer treatment due to the high frequency of dysregulation with this pathway in tumorigenesis^21^,^22^,^23^.

While there is substantial evidence demonstrating that inhibiting PI3Kδ hinders Th17 differentiation, the more clinically relevant scenario is to inhibit cytokine production from committed Th17 cells after differentiation when they have already become pathogenic. In this study, we examined an isoform-specific inhibitor of PI3Kδ *in vitro*, with specific focus on the ability of this compound to inhibit production of Th17 cytokines from effector Th17 cells. We demonstrate that the inhibitors, referred to throughout this paper as Compound A for *in vitro* experiments and Compound B for *in vivo* experiments, effectively inhibits IL-17 production from Th17 cells but also inhibits cytokine production from other Th lineages. We show that the inhibitor affects T cells directly and does not require presence of antigen presenting cells in culture. Additionally, we were able to determine that the mechanism through which it affects cytokine production is through inhibition of the AKT signaling pathway.

## Results

### Inhibition of PI3Kδ with Compound A results in potent, dose-dependent inhibition of IL-17 production from murine Th17 cells

**Figure 1:**
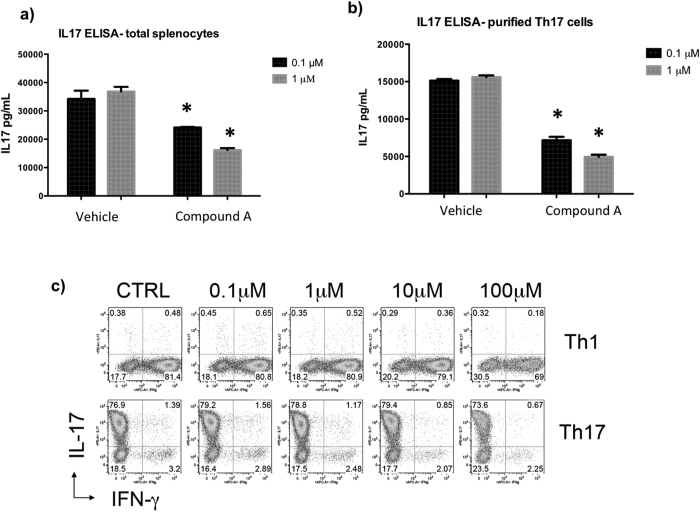
Inhibiting PI3Kδ results in potent, dose-dependent suppression of IL-17 production from total splenocytes and isolated Th17 cells. (**a**) Transgenic OT II mouse total splenocytes, which contain T cells that are specific to ovalbumin peptide, were differentiated into Th17 cells and treated with inhibitor compounds at two different concentrations on day 3 of differentiation. Compared to vehicle control, the PI3Kδ inhibitor results in significant inhibition of IL-17 production in a dose-dependent manner as measured by ELISA (**b**) CD4+/CD62L+ naïve T helper cells were isolated from an IL-17F reporter mouse which uses Thy1.1 expression as a marker for IL-17F production, and then differentiated into Th17 cells. Th17 cells were then further purified by sorting thy1.1+ cells on flow cytometer and then treated with inhibitor compounds on day 3 of differentiation. Compared to vehicle control, the PI3Kδ inhibitor results in significant inhibition of IL-17 production in a dose-dependent manner as measured by ELISA. *p < 0.05, compared to DMSO (**c**) Transgenic OT II mouse total splenocytes, which contain T cells that are specific to ovalbumin peptide, were differentiated into Th1 and Th17 cells and treated with inhibitor compounds at two concentrations on day 1 of differentiation. Effects of the PI3Kδ inhibitor on T cell polarization were analyzed by intracellular staining by FACS.

It has been established by others that inhibition of PI3Kδ results in suppression of Th17 cell differentiation and function and can alleviate inflammation in human and animal models^24^,^25^,^26^,^27^. We analyzed the ability of a selective PI3Kδ inhibitor, Compound A, to inhibit IL-17 production from Th17 cells *in vitro*. Biochemical potency of Compound A (referred as Compound 18 by Patel *et al*.) is 0.4 nM, and selectivity is 200x or better against the other 3 isoforms^28^. When Th17 cells were differentiated from total OT-II splenocytes and treated with this compound on day three of differentiation, the compound resulted in a potent, dose-dependent inhibition of IL-17 production upon CD3/CD28 stimulation. There was statistically significant IL-17 inhibition even at low concentrations of the PI3Kδ inhibitor (Fig. 1a).

Since the p110 delta subunit of PI3K is most highly expressed in leukocytes^17^, and total splenocytes used in Fig. 1a contain bulk splenocytes, we also verified efficacy of Compound A on isolated T cells. When naïve CD4+/CD62L+ T cells were isolated and then differentiated into Th17 cells over three days, the addition of Compound A demonstrated potent and dose-dependent inhibition of IL-17 production compared to DMSO vehicle control (Fig. 1b). To determine if Compound A affects naïve T cell differentiation into Th1 and/or Th17 lineage *in vitro*, we added the inhibitor at various concentrations from Day 0 of T cell polarization. However, the inhibitor seems to have minimal impacts on T cell differentiation (Fig. 1c).

### PI3Kδ inhibitor Compound A inhibits cytokine production from multiple T helper cell types

**Figure 2:**
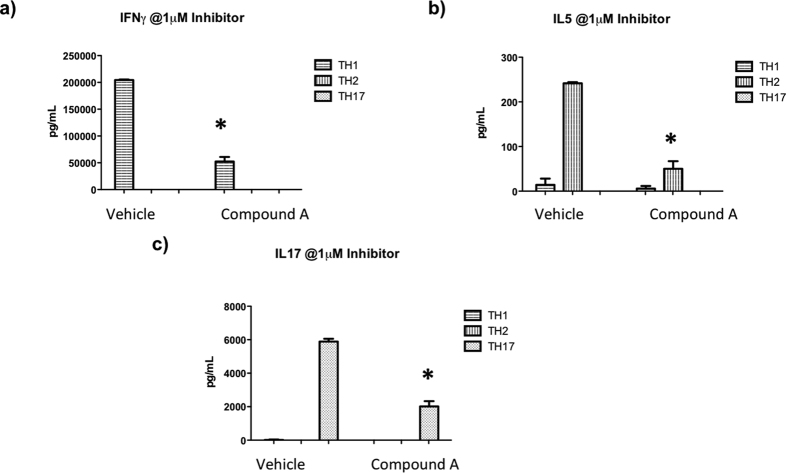
Inhibiting PI3Kδ results in potent, dose-dependent suppression of cytokine production from all three major T helper cell lineages. Transgenic DO.11.10 mouse splenocytes, which contain T cells that are specific to ovalbumin peptide, were differentiated into (**a**) Th1, (**b**) Th2, and (**c**) Th17 cells and treated with inhibitor compounds at 1 μM concentration on day 3 of differentiation. Compared to vehicle control, inhibiting PI3Kδ results in suppression of hallmark cytokine production from all three cell types, indicating that this compound is not specific to Th17 cells. *p < 0.05, compared to DMSO.

To determine whether this PI3Kδ inhibitor was specific to TCR activation in Th17 cells, we differentiated Th1 (IL-2, IL-12, and anti-IL4), Th2 (IL-2, IL-4, and anti-IFNγ), and Th17 (IL-6, TGFβ, IL-23, anti-IL4 and anti-IFNγ) cells *in vitro* for 3 days from naïve CD4 T cells isolated from total splenocytes and treated the cells with the inhibitors upon CD3/CD28 re-stimulation. Luminex on the supernatants of these cells showed significant inhibition of interferon-γ (IFNγ) from Th1 cells, IL-5 from Th2 cells, and continued to show inhibition of IL-17 production from Th17 cells (Fig. 2). Of note, 7-AAD staining was performed on these cells after treatment with Compound A as well as DMSO, and there was no cell toxicity at these concentrations to explain the level of cytokine inhibition (data not shown).

### Compound A directly inhibits AKT function

**Figure 3:**
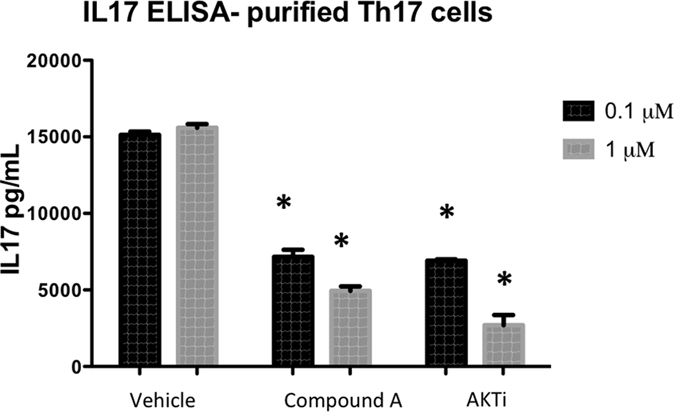
Inhibiting PI3Kδ results in similar suppression of IL-17 production as AKT inhibition from isolated Th17 cells. CD4+/CD62L+ naïve T helper cells were isolated from an IL-17F reporter mouse which uses Thy1.1 expression as a marker for IL-17F production and then differentiated into Th17 cells. Th17 cells were then further isolated by sorting thy1.1+ cells on flow cytometer and then treated with inhibitor compounds on day 3 of differentiation. Compared to vehicle control, the PI3Kδ inhibitor results in strikingly similar inhibition of IL-17 production as an AKT inhibitor. *p < 0.05, compared to DMSO.

Because T cell development and differentiation depends on the canonical pathway for PI3K activation in which PIP3 recruits AKT to the plasma membrane resulting in its phosphorylation and activation^15^, we hypothesized that inhibition of AKT would also result in IL-17 inhibition. Naïve T cells were isolated from mouse splenocytes and treated with Compound A or a commercially available inhibitor of AKT isoforms 1 and 2 on day 3 of differentiation. Cells were then stimulated on day 5 with CD3/CD28 stimulation. We observed inhibition of IL-17 production with both inhibitors (Fig. 3) consistent with the notion that both PI3Kδ and AKT are required for CD3/CD28 stimulated IL-17 production.

**Figure 4:**
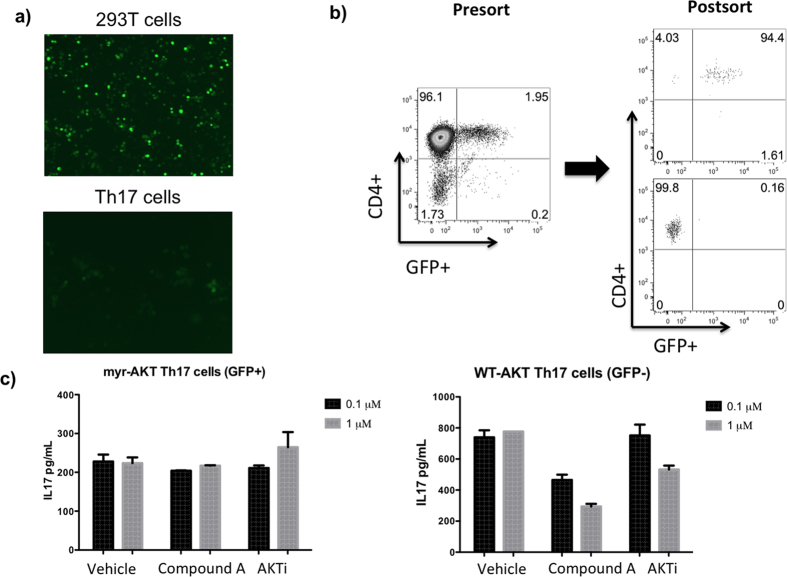
Th17 cells transfected with constitutively active AKT are resistant to effects of PI3Kδ inhibition compared to Th17 cells containing wild type AKT (**a**) 293T cells were transduced with a plasmid containing GFP and myristoylated AKT as well as retroviral packaging plasmid (left). Mouse Th17 cells, derived from OT II splenocytes, were then transfected with retrovirus (right). (**b**) After retroviral transduction, splenocytes were sorted into GFP+ and GFP- populations. (**c**) OT II mouse splenocytes were treated with PI3Kδ inhibitor and AKT inhibitor. GFP+ Th17 cells, which contain constitutively active AKT, do not demonstrate IL-17 inhibition when treated with PI3Kδ inhibitor while GFP- Th17 cells, which contain wild type AKT, continue to demonstrate potent inhibition of IL-17 production. *p < 0.05.

To confirm that this PI3Kδ inhibitor is indeed acting through the AKT pathway, we transduced murine Th17 cells with a retrovirus expressing myristoylated AKT^29^, which is a constitutively active form of AKT (Fig. 4a). Once these Th17 cells were transduced, they were sorted by flow cytometry into a GFP negative population expressing wild type AKT and a GFP positive population expressing myristoylated AKT (Fig. 4b). When these Th17 cells were treated with AKT inhibitor or Compound A prior to CD3/CD28 stimulation, the GFP positive cells that contain constitutively active AKT were resistant to the effects of Compound A, while the GFP negative cells that contain wild type AKT continue to demonstrate potent, dose-dependent inhibition of IL-17 production (Fig. 4c). Taken together, these data demonstrate that the mechanism through which CD3/CD28 stimulated IL-17 production is inhibited in Th17 cells by Compound A is through suppressing AKT activation.

### Compound B is a Potent and Selective Inhibitor of PI3Kδ

A novel selective PI3Kδ inhibitor, Compound B, that had potency, demonstrable oral bioavailability and adequate plasma half-life to evaluate its activity *in vivo* in mice, was utilized. Compound B (GS-649443) is structurally related to Compound A and share the same core (manuscript in preparation). However, Compound B offers superior mouse pharmacokinetics over Compound A, and was therefore preferred for the *in vivo* efficacy studies. In a FRET-based *in vitro* kinase assay, the Compound B inhibited PI3Kδ with an IC_50_ of 0.28 nM and it was 87- to 4150-fold more selective over the other PI3K class I enzymes (manuscript in preparation). In addition, Compound B was also >2500-fold more selective for PI3Kδ over other related kinases, such as CIIα, CIIβ, hVPS34, PI4Kα, mTOR, PIP5K1α, and PIP5K2β (manuscript in preparation).

**Figure 5:**
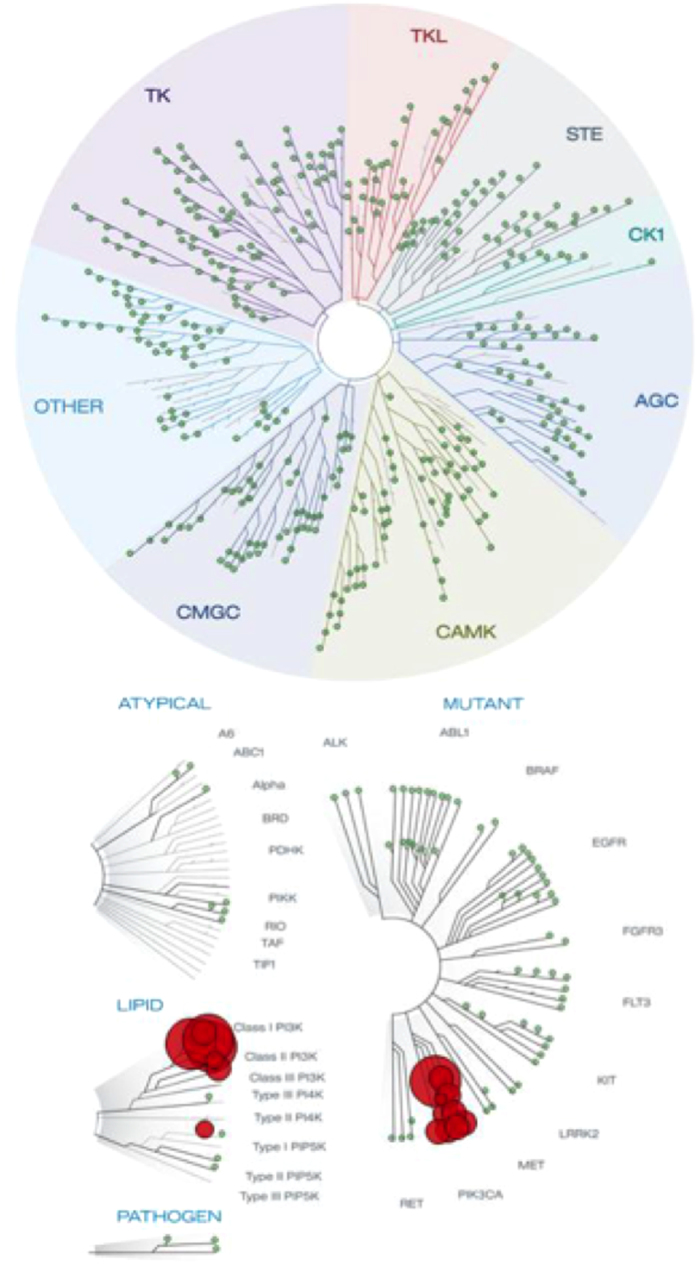
Compound B binds selectively to class I PI3K. Compound B specificity was tested in a kinase-selectivity profiling assay on a panel of 456 kinases. Compound B (10 μM) was tested in an ATP site competition assay. Compound B was considered active for a kinase when the kinase fraction that remained bound to ATP was less than 30%. Red circles indicate the kinases for which Compound B shows binding activity. Compound B showed no significant binding activity outside the PI3K family.

Although Compound B selectivity for the PI3Kδ Class I PI3K isoform was shown by IC_50_ determinations, we tested Compound B binding to kinases outside the PI3K family using a panel of 456 kinases (KinomeScan; DiscoveRx)^30^. A high Compound B concentration (10 μM) in a KinomeScan assay was used. Compound B only binds class I PI3K (Fig. 5, Supplemental Table 1) and inhibits PI3Kδ at an IC_50_ of ∼0.28 nM, further demonstrating a high degree of selectivity for PI3Kδ.

### Compound B inhibits human T-cell cytokine production

**Figure 6:**
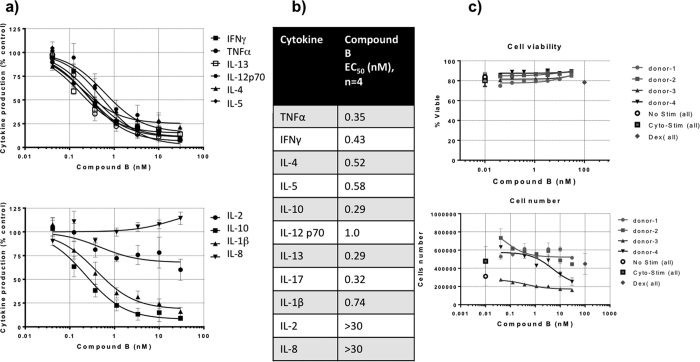
Compound B inhibits Th1 and Th2 cytokine secretion by activated T cells. Cytokines produced by activated T cells were analyzed using human PBMC preincubated with serial dilutions of Compound B (48 h) and activated via anti-CD3/MHC-II (see Methods). Cytokine levels in culture supernatants were assessed using MSD assay plate (Meso Scale Discovery, Gaithersburg, MD). Graphs show percent inhibition of cytokine production at different Compound B concentrations compared to maximum cytokine levels (without Compound B; 100%). The table shows the apparent IC_50_ for the inhibitor.

The ability of Compound B to inhibit TCR-stimulated cytokine release was evaluated in normal human PBMCs. Isolated PBMCs in serum-free AIM-V media were treated with either vehicle or Compound B followed by stimulation by CytoStim reagent. The production of cytokines was evaluated by Meso Scale Discovery assays after 48 hours of stimulation. Compound B inhibited TNFα, IFNγ, IL-4, IL-5, IL-10, IL-12 p70, IL-13, IL-17, and IL-1β secretion in human PBMCs with geometric mean EC50 values of 0.29 to 1.0 nM, demonstrating that Compound B inhibits TCR-triggered signaling that leads to secretion of these inflammatory cytokines (Fig. 6a). Production of IL-2 was only modestly inhibited and IL-8 production was not inhibited. The individual EC_50_ values are listed (Fig. 6b) and these values are much lower that the EC50 values (Table 1) of Idelalisib (GS-1101, CAL-101), a potent and selective small molecule inhibitor of the delta isoform of the p110 subunit PI3K (PI3K p110δ) recently approved for the treatment of chronic lymphocytic leukemia and non-Hodgkin lymphoma^31^,^32^,^33^. Cell viability from 4 donors was not significant altered by increasing the concentration of compound B (Fig. 6c). These data suggest that Compound B is more potent than Idelalisib. This is of clinical significance as increasing the potency could reduce the dose level and may limit dose-associated toxicities.

### Inhibition of PI3Kδ results in reduced IL-17 mediated inflammation

**Figure 7:**
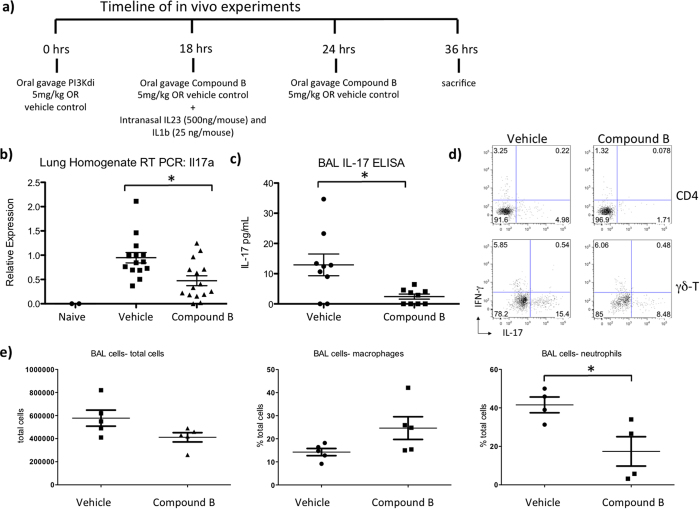
*In vivo* Inhibition of PI3Kδ in mice results in decreased IL-17 production by Gamma Delta T cells. (**a**) B6 mice were treated with Compound B or vehicle control by oral gavage. All mice received intranasal IL-1b and IL-23 to activate γδ T cells at indicated time points. (**b)** Real time PCR demonstrates a significant reduction in IL-17a expression in the lung homogenate of mice treated with Compound B. Naïve mice that did not receive intranasal IL-1b or IL-23 did not express any IL-17a. This is pooled data from 3 independent experiments. (**c)** IL-17 ELISA shows a significantly decreased IL-17 levels in the BAL fluid in mice treated with Compound B. This is pooled data from 2 independent experiments. (**d)** Representative intracellular cytokine staining FACS plots for IL-17 and IFN-γ gated on both γδ-T cells and CD4^+^ T cells from the lung mononuclear cells were shown (N = 4–5). (**e**) Flow cytometry showed a significantly lower percentage of neutrophils in the BAL fluid of mice treated with experimental compound while there were no significant differences in total cells or percentage of macrophages between groups. This data is representative of 3 independent experiments.

While PI3Kδ is known to play an important role in IL-17 production by CD4+ T cells, its role in IL-17 production by γδ T cells is less clear. However, γδ T cells have been shown to play a significant role in the pathogenesis of autoimmune diseases^34^, and thus inhibiting PI3Kδ could be therapeutically beneficial if it does result in inhibition of γδ T cell function. We sought to determine whether inhibiting PI3Kδ in pulmonary γδ T cells could also result in decreased IL-17 production. To examine this we administered Compound B by oral gavage to mice that were treated with intranasal IL-1β and IL-23 which activates pulmonary γδ T cells to express IL-17 (Fig. 7a)^35^,^36^. The lungs of mice that were treated with the experimental compound demonstrated 50% reduced expression of IL-17A by real time PCR compared with mice that received vehicle control (Fig. 7b). Additionally, there was also 50% reduction of pulmonary IL-17 at the protein level in broncheoalveolar lavage (BAL) fluid of mice treated with the PI3Kδ inhibitor (Fig. 7c). To further evaluate the effect of the experimental compound on the inflammatory cell infiltrate in the lung alveolar space, we performed FACS analysis on the BAL cells. Consistent with suppression of IL-17, neutrophils were selectively decreased as we expected in the BAL fluid of mice treated with Compound B while percentages of macrophages and total cells in the BAL fluid were not statistically different between groups (Fig. 7d). To determined the cellular sources of IL-17, we performed intracellular cytokine staining on the mononuclear cells isolated from the lungs of mice received the treatment of Compound B. Both γδ-T and CD4^+^ T cells appeared to be IL-17 producers in this model and the IL-17 production was suppressed by Compound B in both cell populations (Fig. 7e).

## Discussion

In this study, we have shown that Compound A, a novel isoform-specific inhibitor of PI3Kδ, has a potent and dose-dependent inhibitory effect on cytokine production from T helper cells when they are stimulated through TCR and CD28 activation. It inhibited IL-17 production from Th17 cells, and also inhibited production of key cytokines by murine and human Th1 and Th2 cells after stimulation. This non-specific effect on all three major T helper cell subsets is not surprising, as there is no evidence that the delta isoform of PI3K is differentially expressed among various T helper cell lineages. In fact, the broad-spectrum suppression of various Th effectors may be beneficial in clinical setting since many inflammatory diseases such as severe asthma and rheumatoid arthritis are characterized by combinations of Th1, Th2 and/or Th17 driven immune responses^37^,^38^.

The mechanism by which Compound A inhibited cytokine production was through attenuating the AKT signaling pathway. This pathway is known to regulate differentiation of Th17 cells by AKT activation of the Mechanistic Target of Rapamycin Complex 1 (mTORC1), thereby activating HIF-1α and S6K2, as well as inhibiting Gfi1^16^,^18^,^20^. There is some debate over whether inhibition of PI3Kδ affects Th17 differentiation by inducing FoxP3 expression, but the evidence supporting this mechanism is mainly found in experiments where PI3Kδ expression is modified prior to T helper cell differentiation^18^,^19^,^39^,^40^. For the purposes of this paper, we focused on inhibiting PI3Kδ after differentiation of these effector T helper cells, as this is more analogous to true disease states in which effector T cells have already formed.

Since γδ T cells have been implicated in the pathogenesis of autoimmune diseases^34^, evaluation as to whether PI3Kδ inhibition could inhibit IL-17 production from γδ T cells would be impactful. We used experimental Compound B, which is a PI3Kδ inhibitor structurally-related to Compound A, with more suitable pharmacokinetic properties for an *in vivo* mouse model of pulmonary inflammation. This study demonstrates that pulmonary γδ T cell function is affected by PI3Kδ inhibition in that IL-17 was decreased following stimulation with IL-1β and IL-23 and this results in reduced neutrophil infiltrates in BAL fluid. This is likely a reflection of decreased neutrophil recruitment to the lung in these mice treated with experimental compound, as inhibition of IL-17 may result in attenuated activation of its downstream chemokines, which are responsible for neutrophil recruitment^41^. There was a trend toward decreased expression of *Cxcl1* and *Csf3* mRNA in the mice treated with experimental compound, but this data did not reach statistical significance (data not shown). Since CXCL1 acts as a neutrophil chemoattractant while G-CSF plays more of a role in neutrophil mobilization, the ultimate effect of decreased neutrophil recruitment in this experiment may have been additive or synergistic from the combination of the effects of decreased *Cxcl1* and *Csf3*^42^,^43^,^44^.

Mutations and dysregulation in the PI3K/AKT pathways are heavily involved in cancer pathogenesis, and thus this pathway is highly targeted in cancer research. The PI3Kδ isoform specifically has been shown to be abnormally expressed or abnormally signal in several malignancies and small molecule inhibitors of this isoform are in clinical trials for cancer treatment and one is FDA approved for the treatment of CLL^23^,^45^. However, given the preferential expression of this isoform in leukocytes, it is more recently being investigated as a therapeutic target in nonmalignant inflammatory conditions as well. Inhibition of PI3Kδ is known to suppress Th2 inflammatory responses from PBMCs taken from human patients with environmental allergies and suppresses IFNγ, IL-17, and TNF-α production by PBMCs from human patients with rheumatoid arthritis^26^. PI3Kδ kinase-dead knock-in mice demonstrate reduced skin inflammation in psoriasis models, and PBMCs from human psoriasis patients have lower ex vivo stimulated IL-17 production when treated with PI3Kδ inhibitors in cell culture^27^. A gain-of-function mutation in the PI3Kδ isoform has also been discovered which is associated with recurrent respiratory infections and airway damage, highlighting the importance of this molecule in inflammatory diseases^46^.

There are currently a myriad of treatment options available for patients with autoimmune inflammatory conditions including antimetabolites, calcineurin inhibitors, monoclonal antibodies to specific cytokines and their receptors, B cell inhibitors, and even alkylating agents. However, for all of the successes that each therapy has experienced, there are always patients who are refractory and require change to or addition of other agents. Therefore, there is a need for new classes of medications for the treatment of autoimmune inflammatory conditions; PI3Kδ inhibitors could have an important role in the treatment of these conditions and these molecules are currently being evaluated for treatment of several inflammatory disorders.

## Materials and Methods

### Animals

4–8 week old female OT-II and DO11.10 mice were obtained from Jackson Lab (Bar Harbor, ME). Thy1.1 IL-17F reporter mice, originally developed in the lab of Casey Weaver at University of Alabama Birmingham^47^, were bred in-house at The University of Pittsburgh. All animal studies were approved by the Institutional Animal Care and Use Committee (IACUC) of the University of Pittsburgh. All experiments were performed in accordance with IACUC guidelines and regulations. 8–10 week old female C57BL/6 mice from Jackson lab were used for *in vivo* studies. On day 0 they were given 5mg/kg Compound B) by oral gavage or same volume of vehicle control. Compound B or vehicle control was dosed every 12–18 hours for 3 doses on day 0 and day 1. On day 1, 24 hours before sacrificing, every mouse received Intranasal IL-23 (500 ng/mouse) and IL-1β (25 ng/mouse) to induce gamma-delta T cell activation^35^,^36^. On Day 2 the mice were sacrificed, BAL was performed, and lungs were harvested. All animal studies were approved by the Institutional Animal Care and Use Committee of the University of Pittsburgh.

### T cell differentiation

Total splenocytes or naive CD4+/CD62L+ cells were incubated for 3 days in the presence of IL-2 (50 units/mL), IL-12(10 ng/mL), and anti-IL4(5 μg/mL) for Th1 cell differentiation; IL-2 (50 units/mL), IL-4 (10 ng/mL), and anti-IFNγ (5 μg/mL) for Th2 cell differentiation; or IL-6(40 ng/mL), TGFβ(5 ng/mL), IL-23(10 ng/mL), anti-IL4(5 μg/mL) and anti-IFNγ (5 μg/mL) for Th17 cell differentiation. All recombinant mouse cytokines were purchased from R&D Systems, Inc (San Diego, CA) and eBioscience. OT-II and DO11.10 splenocytes were activated by chicken ovalbumin peptide, OVA (323–339), (AnaSpec, Fremont, CA) and naïve CD4+/CD62L+ T cells were activated by plate-bound anti-CD3 and anti-CD28 antibodies from eBioscience, Inc. Naïve CD4+/CD62L+ T cells were isolated by MACS beads from Miltenyi Biotec (Auburn, CA).

### Cytokine Inhibitor Compounds

Compounds were reconstituted at 10 mM with DMSO and diluted to 1 μM, and 0.1 μM for cell culture. Cells are washed twice prior to addition of inhibitors. 30–60 minutes after addition of inhibitors, T cells were re-stimulated with anti-CD3/anti-CD28 coated beads (Life Technologies, Grand Island, NY). Cells are incubated with inhibitor compounds and beads for 18–24 hours. AKT 1/2 inhibitor (cat# 612847-09-03) was purchased from EMD Millipore (Billerica, MA).

### T cell transduction

Th17 cells were differentiated from OT-II splenocytes while 293T cells were transduced with myrAKT plasmid and pCL-ECO packaging plasmid (Biomol, Hamburg, Germany) using Polyethyleneimine and NaCl. On day 3 of 293T cell transduction and Th17 cell differentiation, retroviral supernatant was harvested and Th17 cells were centrifuged in a 24-well plate with 1 mL viral supernatant/well and polybrene 10 μg/mL at 300 g for 1 hr. On day 4, retroviral supernatant was harvested again and centrifugation repeated. Th17 cells were restimulated with chicken ovalbumin peptide after each round of transduction, then incubated in IMDM with TGFβ, IL-6, and IL-23 for 5 additional days. Th17 cells were then sorted into CD4+/GFP+ and CD4+/GFP- populations.

### Statistics

All data are presented as the mean ± SEM. For comparison between two groups, student *t*-test was used. A P-value of less than 0.05 was considered significant.

## Additional Information

**How to cite this article**: Way, E. E. *et al*. Dose-Dependent Suppression of Cytokine production from T cells by a Novel Phosphoinositide 3-Kinase Delta Inhibitor. *Sci. Rep.* **6**, 30384; doi: 10.1038/srep30384 (2016).

## Supplementary Material

Supplementary Information

## Figures and Tables

**Table 1:** *In Vitro* Potency of Idelalisib on Human T-Cell Cytokine Production.

**Cell Type**	**Stimuli (Receptor)**	**Assay**	**Readout**	**EC**_**50**_**(nM)**
T cells	Anti-CD3/Anti-MHC II (TCR/MHC II)	Cytokines release	IL-4	2.4
			IL-5	2.7
			IL-13	3.1
			IL-17	3.5
			TNFα	5.2
			IFNγ	4.8
			IL-1β	6.1
			IL-12p70	2.3
			IL-10	2.6
