# Supplementary material for: Dose-Dependent Suppression of Cytokine production from T cells by a Novel Phosphoinositide 3-Kinase Delta Inhibitor
Source: Sci Rep. 2016 Jul 27;6:30384. doi: 10.1038/srep30384 (PMC4961957; doi:10.1038/srep30384)
Supplement: Supplementary Information [file srep30384-s1.doc]

**Supplementary information:**

**Dose-Dependent Suppression of Cytokine production from T cells by a Novel Phosphoinositide 3-Kinase Delta Inhibitor**

Emily E. Way^1^, Giraldina Trevejo-Nunez^1^, Lawrence P. Kane^2^, Bart H. Steiner^3^, Kamal D. Puri^3^, and Jay K. Kolls^1^*, Kong Chen^1^

^1^Richard King Mellon Foundation Institute for Pediatric Research, Children’s Hospital of Pittsburgh, Pittsburgh, PA

^2^Department of Immunology, University of Pittsburgh, Pittsburgh, PA

^3^Gilead Sciences, Inc. Seattle, WA

To whom correspondence should be addressed:

Dr. Jay K. Kolls, Department of Pediatrics, Children's Hospital of Pittsburgh of University of Pittsburgh Medical Center, Rangos Research Building, 4401 Penn Avenue, Pittsburgh, PA 15224

Phone: (412) 692-7710, Fax: (412) 692-7636, E-mail: [Jay.Kolls@chp.edu](mailto:Jay.Kolls@chp.edu)

**Supplemental Table 1: Compound B - in vitro kinase profiling.** Kinase selectivity profiling for Compound B was tested at 10 μM in ATP site-dependent competition binding assays for 456 kinases as described in methods. The table indicates the percent of enzyme that remained bound to the immobilized ATP probe in the presence of Compound B.

Supplemental Table 1

| **Ambit Gene Symbol** | **Percent Binding** | **Ambit Gene Symbol** | **Percent Binding** |
| --- | --- | --- | --- |
| AAK1 | 98 | AURKB | 100 |
| ABL1(E255K)-phosphorylated | 90 | AURKC | 82 |
| ABL1(F317I)-nonphosphorylated | 88 | AXL | 100 |
| ABL1(F317I)-phosphorylated | 68 | BIKE | 91 |
| ABL1(F317L)-nonphosphorylated | 92 | BLK | 84 |
| ABL1(F317L)-phosphorylated | 93 | BMPR1A | 85 |
| ABL1(H396P)-nonphosphorylated | 100 | BMPR1B | 79 |
| ABL1(H396P)-phosphorylated | 92 | BMPR2 | 99 |
| ABL1(M351T)-phosphorylated | 83 | BMX | 89 |
| ABL1(Q252H)-nonphosphorylated | 100 | BRAF | 94 |
| ABL1(Q252H)-phosphorylated | 100 | BRAF(V600E) | 98 |
| ABL1(T315I)-nonphosphorylated | 92 | BRK | 100 |
| ABL1(T315I)-phosphorylated | 90 | BRSK1 | 89 |
| ABL1(Y253F)-phosphorylated | 95 | BRSK2 | 61 |
| ABL1-nonphosphorylated | 100 | BTK | 88 |
| ABL1-phosphorylated | 82 | BUB1 | 79 |
| ABL2 | 96 | CAMK1 | 98 |
| ACVR1 | 80 | CAMK1D | 100 |
| ACVR1B | 92 | CAMK1G | 100 |
| ACVR2A | 93 | CAMK2A | 93 |
| ACVR2B | 100 | CAMK2B | 94 |
| ACVRL1 | 100 | CAMK2D | 96 |
| ADCK3 | 92 | CAMK2G | 100 |
| ADCK4 | 69 | CAMK4 | 73 |
| AKT1 | 94 | CAMKK1 | 100 |
| AKT2 | 83 | CAMKK2 | 91 |
| AKT3 | 87 | CASK | 97 |
| ALK | 87 | CDC2L1 | 79 |
| ALK(C1156Y) | 82 | CDC2L2 | 100 |
| ALK(L1196M) | 83 | CDC2L5 | 99 |
| AMPK-alpha1 | 84 | CDK11 | 100 |
| AMPK-alpha2 | 86 | CDK2 | 83 |
| ANKK1 | 84 | CDK3 | 100 |
| ARK5 | 94 | CDK4-cyclinD1 | 97 |
| ASK1 | 80 | CDK4-cyclinD3 | 100 |
| ASK2 | 83 | CDK5 | 90 |
| AURKA | 58 | CDK7 | 90 |
| CDK8 | 100 | EGFR(G719S) | 98 |
| CDK9 | 91 | EGFR(L747-E749del, A750P) | 95 |
| CDKL1 | 88 | EGFR(L747-S752del, P753S) | 73 |
| CDKL2 | 100 | EGFR(L747-T751del,Sins) | 100 |
| CDKL3 | 100 | EGFR(L858R) | 97 |
| CDKL5 | 100 | EGFR(L858R,T790M) | 100 |
| CHEK1 | 72 | EGFR(L861Q) | 67 |
| CHEK2 | 100 | EGFR(S752-I759del) | 69 |
| CIT | 100 | EGFR(T790M) | 48 |
| CLK1 | 88 | EIF2AK1 | 90 |
| CLK2 | 94 | EPHA1 | 98 |
| CLK3 | 100 | EPHA2 | 100 |
| CLK4 | 80 | EPHA3 | 81 |
| CSF1R | 99 | EPHA4 | 100 |
| CSF1R-autoinhibited | 100 | EPHA5 | 86 |
| CSK | 85 | EPHA6 | 86 |
| CSNK1A1 | 100 | EPHA7 | 95 |
| CSNK1A1L | 100 | EPHA8 | 95 |
| CSNK1D | 76 | EPHB1 | 94 |
| CSNK1E | 92 | EPHB2 | 100 |
| CSNK1G1 | 78 | EPHB3 | 89 |
| CSNK1G2 | 64 | EPHB4 | 95 |
| CSNK1G3 | 61 | EPHB6 | 58 |
| CSNK2A1 | 92 | ERBB2 | 100 |
| CSNK2A2 | 41 | ERBB3 | 100 |
| CTK | 73 | ERBB4 | 100 |
| DAPK1 | 90 | ERK1 | 92 |
| DAPK2 | 100 | ERK2 | 84 |
| DAPK3 | 100 | ERK3 | 87 |
| DCAMKL1 | 81 | ERK4 | 100 |
| DCAMKL2 | 92 | ERK5 | 94 |
| DCAMKL3 | 99 | ERK8 | 83 |
| DDR1 | 97 | ERN1 | 62 |
| DDR2 | 83 | FAK | 98 |
| DLK | 96 | FER | 98 |
| DMPK | 91 | FES | 90 |
| DMPK2 | 100 | FGFR1 | 97 |
| DRAK1 | 100 | FGFR2 | 87 |
| DRAK2 | 98 | FGFR3 | 100 |
| DYRK1A | 100 | FGFR3(G697C) | 91 |
| DYRK1B | 97 | FGFR4 | 98 |
| DYRK2 | 90 | FGR | 96 |
| EGFR | 100 | FLT1 | 93 |
| EGFR(E746-A750del) | 72 | FLT3 | 98 |
| EGFR(G719C) | 84 | FLT3(D835H) | 100 |
| FLT3(D835Y) | 100 | KIT(D816V) | 100 |
| FLT3(ITD) | 98 | KIT(L576P) | 86 |
| FLT3(K663Q) | 89 | KIT(V559D) | 100 |
| FLT3(N841I) | 47 | KIT(V559D,T670I) | 99 |
| FLT3(R834Q) | 94 | KIT(V559D,V654A) | 100 |
| FLT3-autoinhibited | 67 | KIT-autoinhibited | 91 |
| FLT4 | 100 | LATS1 | 65 |
| FRK | 100 | LATS2 | 74 |
| FYN | 82 | LCK | 93 |
| GAK | 91 | LIMK1 | 94 |
| GCN2(Kin.Dom.2,S808G) | 100 | LIMK2 | 60 |
| GRK1 | 100 | LKB1 | 93 |
| GRK4 | 98 | LOK | 93 |
| GRK7 | 100 | LRRK2 | 100 |
| GSK3A | 85 | LRRK2(G2019S) | 92 |
| GSK3B | 97 | LTK | 88 |
| HASPIN | 100 | LYN | 100 |
| HCK | 100 | LZK | 100 |
| HIPK1 | 82 | MAK | 76 |
| HIPK2 | 84 | MAP3K1 | 97 |
| HIPK3 | 94 | MAP3K15 | 95 |
| HIPK4 | 79 | MAP3K2 | 95 |
| HPK1 | 94 | MAP3K3 | 94 |
| HUNK | 97 | MAP3K4 | 77 |
| ICK | 86 | MAP4K2 | 92 |
| IGF1R | 88 | MAP4K3 | 100 |
| IKK-alpha | 95 | MAP4K4 | 100 |
| IKK-beta | 100 | MAP4K5 | 96 |
| IKK-epsilon | 74 | MAPKAPK2 | 91 |
| INSR | 43 | MAPKAPK5 | 100 |
| INSRR | 92 | MARK1 | 100 |
| IRAK1 | 76 | MARK2 | 98 |
| IRAK3 | 94 | MARK3 | 95 |
| IRAK4 | 56 | MARK4 | 100 |
| ITK | 97 | MAST1 | 85 |
| JAK1(JH1domain-catalytic) | 93 | MEK1 | 96 |
| JAK1(JH2domain-pseudokinase) | 100 | MEK2 | 100 |
| JAK2(JH1domain-catalytic) | 100 | MEK3 | 93 |
| JAK3(JH1domain-catalytic) | 65 | MEK4 | 96 |
| JNK1 | 87 | MEK5 | 97 |
| JNK2 | 95 | MEK6 | 94 |
| JNK3 | 92 | MELK | 83 |
| KIT | 90 | MERTK | 89 |
| KIT(A829P) | 81 | MET | 78 |
| KIT(D816H) | 100 | MET(M1250T) | 83 |
| MET(Y1235D) | 80 | PAK3 | 76 |
| MINK | 85 | PAK4 | 100 |
| MKK7 | 100 | PAK6 | 100 |
| MKNK1 | 99 | PAK7 | 90 |
| MKNK2 | 94 | PCTK1 | 86 |
| MLCK | 100 | PCTK2 | 84 |
| MLK1 | 94 | PCTK3 | 94 |
| MLK2 | 98 | PDGFRA | 92 |
| MLK3 | 97 | PDGFRB | 95 |
| MRCKA | 100 | PDPK1 | 100 |
| MRCKB | 98 | PFCDPK1(P.falciparum) | 100 |
| MST1 | 94 | PFPK5(P.falciparum) | 86 |
| MST1R | 69 | PFTAIRE2 | 97 |
| MST2 | 79 | PFTK1 | 100 |
| MST3 | 98 | PHKG1 | 93 |
| MST4 | 100 | PHKG2 | 85 |
| MTOR | 82 | PIK3C2B | 7.2 |
| MUSK | 98 | PIK3C2G | 1.6 |
| MYLK | 88 | PIK3CA | 1.7 |
| MYLK2 | 84 | PIK3CA(C420R) | 2.6 |
| MYLK4 | 93 | PIK3CA(E542K) | 3.8 |
| MYO3A | 98 | PIK3CA(E545A) | 2.6 |
| MYO3B | 63 | PIK3CA(E545K) | 3.9 |
| NDR1 | 98 | PIK3CA(H1047L) | 2 |
| NDR2 | 84 | PIK3CA(H1047Y) | 2.8 |
| NEK1 | 93 | PIK3CA(I800L) | 27 |
| NEK10 | 100 | PIK3CA(M1043I) | 0 |
| NEK11 | 100 | PIK3CA(Q546K) | 4 |
| NEK2 | 100 | PIK3CB | 0 |
| NEK3 | 88 | **PIK3CD** | **0** |
| NEK4 | 100 | PIK3CG | 0 |
| NEK5 | 89 | PIK4CB | 49 |
| NEK6 | 85 | PIM1 | 81 |
| NEK7 | 78 | PIM2 | 94 |
| NEK9 | 95 | PIM3 | 98 |
| NIK | 88 | PIP5K1A | 79 |
| NIM1 | 100 | PIP5K1C | 6 |
| NLK | 66 | PIP5K2B | 59 |
| OSR1 | 65 | PIP5K2C | 64 |
| p38-alpha | 94 | PKAC-alpha | 86 |
| p38-beta | 88 | PKAC-beta | 94 |
| p38-delta | 80 | PKMYT1 | 95 |
| p38-gamma | 86 | PKN1 | 88 |
| PAK1 | 98 | PKN2 | 90 |
| PAK2 | 93 | PKNB(M.tuberculosis) | 58 |
| PLK1 | 84 | RSK4(Kin.Dom.2-C-terminal) | 90 |
| PLK2 | 100 | S6K1 | 93 |
| PLK3 | 79 | SBK1 | 70 |
| PLK4 | 67 | SGK | 74 |
| PRKCD | 99 | SgK110 | 78 |
| PRKCE | 100 | SGK2 | 65 |
| PRKCH | 83 | SGK3 | 85 |
| PRKCI | 92 | SIK | 83 |
| PRKCQ | 97 | SIK2 | 99 |
| PRKD1 | 92 | SLK | 89 |
| PRKD2 | 93 | SNARK | 100 |
| PRKD3 | 64 | SNRK | 90 |
| PRKG1 | 90 | SRC | 93 |
| PRKG2 | 98 | SRMS | 100 |
| PRKR | 38 | SRPK1 | 91 |
| PRKX | 80 | SRPK2 | 100 |
| PRP4 | 93 | SRPK3 | 100 |
| PYK2 | 100 | STK16 | 100 |
| QSK | 77 | STK33 | 100 |
| RAF1 | 76 | STK35 | 96 |
| RET | 99 | STK36 | 91 |
| RET(M918T) | 100 | STK39 | 98 |
| RET(V804L) | 96 | SYK | 98 |
| RET(V804M) | 100 | TAK1 | 89 |
| RIOK1 | 82 | TAOK1 | 79 |
| RIOK2 | 67 | TAOK2 | 86 |
| RIOK3 | 100 | TAOK3 | 83 |
| RIPK1 | 100 | TBK1 | 92 |
| RIPK2 | 85 | TEC | 89 |
| RIPK4 | 84 | TESK1 | 98 |
| RIPK5 | 86 | TGFBR1 | 84 |
| ROCK1 | 93 | TGFBR2 | 92 |
| ROCK2 | 100 | TIE1 | 89 |
| ROS1 | 100 | TIE2 | 100 |
| RPS6KA4(Kin.Dom.1-N-terminal) | 85 | TLK1 | 89 |
| RPS6KA4(Kin.Dom.2-C-terminal) | 98 | TLK2 | 98 |
| RPS6KA5(Kin.Dom.1-N-terminal) | 88 | TNIK | 90 |
| RPS6KA5(Kin.Dom.2-C-terminal) | 100 | TNK1 | 96 |
| RSK1(Kin.Dom.1-N-terminal) | 89 | TNK2 | 92 |
| RSK1(Kin.Dom.2-C-terminal) | 90 | TNNI3K | 100 |
| RSK2(Kin.Dom.1-N-terminal) | 100 | TRKA | 90 |
| RSK2(Kin.Dom.2-C-terminal) | 71 | TRKB | 90 |
| RSK3(Kin.Dom.1-N-terminal) | 85 | TRKC | 98 |
| RSK3(Kin.Dom.2-C-terminal) | 100 | TRPM6 | 93 |
| RSK4(Kin.Dom.1-N-terminal) | 100 | TSSK1B | 85 |
| TTK | 77 | WEE2 | 98 |
| TXK | 100 | WNK1 | 100 |
| TYK2(JH1domain-catalytic) | 100 | WNK3 | 92 |
| TYK2(JH2domain-pseudokinase) | 100 | YANK1 | 100 |
| TYRO3 | 78 | YANK2 | 79 |
| ULK1 | 74 | YANK3 | 84 |
| ULK2 | 94 | YES | 92 |
| ULK3 | 57 | YSK1 | 94 |
| VEGFR2 | 81 | YSK4 | 55 |
| VRK2 | 100 | ZAK | 96 |
| WEE1 | 100 | ZAP70 | 100 |
